# Do patients with diabetes with new onset acute myocardial infarction present with different symptoms than non-diabetic patients?

**DOI:** 10.3389/fcvm.2024.1324451

**Published:** 2024-01-15

**Authors:** Timo Schmitz, Bastian Wein, Philip Raake, Margit Heier, Annette Peters, Jakob Linseisen, Christa Meisinger

**Affiliations:** ^1^ Epidemiology, Medical Faculty, University of Augsburg, Augsburg, Germany; ^2^ Department of Cardiology, Respiratory Medicine and Intensive Care, University Hospital Augsburg, Augsburg, Germany; ^3^ KORA Study Centre, University Hospital of Augsburg, Augsburg, Germany; ^4^ Helmholtz Zentrum München, German Research Center for Environmental Health, Institute for Epidemiology, Neuherberg, Germany; ^5^ Chair of Epidemiology, Institute for Medical Information Processing, Biometry and Epidemiology, Medical Faculty, Ludwig-Maximilians-Universität München, Munich, Germany; ^6^ German Center for Diabetes Research (DZD) Neuherberg, Neuherberg, Germany

**Keywords:** myocardial infarction, acute symptoms, diabetes mellitus, atypical presentation, chest pain, shortness of breath

## Abstract

**Background:**

The objective of this study was to investigate the differences in presenting symptoms between patients with and without diabetes being diagnosed with an acute myocardial infarction (AMI).

**Methods:**

A total of 5,900 patients with a first-time AMI were included into the analysis. All patients aged between 25 and 84 years were recorded by the population-based Myocardial Infarction Registry in Augsburg, Germany, between 2010 and 2017. The presence (yes/no) of 12 AMI typical symptoms during the acute event was assessed within the scope of a face-to-face interview. Multivariable adjusted logistic regression models were calculated to analyze the associations between presenting symptoms and diabetes mellitus in AMI patients.

**Results:**

Patients with diabetes had significantly less frequent typical pain symptoms, including typical chest pain. Also, other symptoms like sweating, vomiting/nausea, dizziness/vertigo and fear of death/feeling of annihilation occurred significantly more likely in non-diabetic patients. The only exception was the symptom of shortness of breath, which was found significantly more often in patients with diabetes. In multivariable-adjusted regression models, however, the observed effects were attenuated. In patients younger than 55 years, the associations between diabetes and various symptoms were mainly missing.

**Conclusions:**

Type 2 diabetes mellitus is a risk factor not only for the development of AMI, but is also associated with an adverse outcome after AMI. Atypical clinical presentation additionally complicates the diagnostic process. It is therefore essential for physicians to be aware of the more often atypical symptoms that diabetic AMI patients report.

## 1 Background

Type 2 diabetes is a rising health problem worldwide (1). Among many other health consequences, it increases the risk of coronary artery disease (CAD) with subsequent acute myocardial infarction (AMI) (2). In AMI patients, a diabetes diagnosis additionally goes along with worse outcomes and higher mortality (3, 4). Therefore, early diagnosis and timely treatment of diabetes are especially important. Prior studies indicated that AMI patients with diabetes less often report typical chest pain symptoms and some other typical AMI symptoms (5–10). Thus, diagnostics of AMI in diabetic patients would be impeded. However, there are also studies reporting conflicting results by indicating no (relevant) differences in typical AMI symptoms between patients with and without diabetes (11–15). Consequently, the current scientific knowledge is quite ambiguous and often based on smaller samples of AMI patients. So the aim of this study was to investigate the association between the presence of type 2 diabetes and the frequency of a variety of specific AMI symptoms using population-based data and calculating multivariable logistic regression models thereby taking into account various potential confounders.

## 2 Methods

### 2.1 Study population

This study uses data from the population-based Augsburg Myocardial Infarction Registry. As part of the MONICA-project (Monitoring Trends and Determinants in Cardiovascular disease) it was established in 1984 and since 1996 operated as the KORA Myocardial Infarction Registry (16). Since 2021, it is continued as Augsburg Myocardial Infarction Register at the University Hospital Augsburg. The study area includes the city of Augsburg, Germany, and the two adjacent counties (approximately 680,000 inhabitants in total). All cases of hospitalized AMI were recorded on the following conditions: the patient survived the first 24 h after hospital admission, had his/her primary residence within the study area and was between 25 and 84 years of age at time of infarction. Detailed information on case identification, diagnostic classification of events and quality control of the data can be found in a previous publication (16). All study participants gave written informed consent. Methods of data collection have been approved by the ethics committee of the Bavarian Medical Association (Bayerische Landesärztekammer) and the study was performed in accordance with the Declaration of Helsinki.

For the following statistical analysis, only patients with a first-time AMI between 2010 and 2017 were considered (*n* = 6,327 cases). All patients with missing information on typical chest pain symptoms or relevant covariables (*n* = 427) were excluded. The final study population consisted of 5,900 patients with incident AMI.

### 2.2 Data collection

During hospital stay the patients were interviewed by trained study nurses in a face-to-face interview at the general ward. A standardized questionnaire was used including questions about specific symptoms in the context of the acute event. The patients were asked about the presence (yes/no) of the following symptoms: typical chest pain symptoms (defined as “chest pain or a feeling of pressure or tightness behind the breastbone”), pain in the left arm/shoulder, pain in the right arm/shoulder, pain between the shoulder blades, pain in the upper abdomen, pain in the throat/jaw, sweating, vomiting/nausea, shortness of breath, dizziness/vertigo, syncope/unconsciousness and fear of death/feeling of annihilation. In addition to the interview, the patients’ medical chart was reviewed in order to collect demographic data, data on cardiovascular risk factors, medical history, comorbidities, laboratory values, in-hospital course and medication.

The presence of diabetes mellitus was assessed by the patient's statement in the interview as well as by extracting relevant information from the medical record. The patient was assigned to the diabetes group if either he clearly stated to have a medical diagnosis of diabetes mellitus and/or there was an explicit indication of a diabetes mellitus diagnosis in the medical chart. In case neither in the interview nor in the medical chart there was an explicit indication of diabetes mellitus, the patients was assigned to the non-diabetes group. For this study, we did not distinguish between diabetes mellitus type 1 and type 2.

### 2.3 Statistical analysis

Categorical variables were presented as absolute frequencies with percentages and Chi-square tests were used to test for group differences. Continuous variables were presented as mean (standard deviation, SD) or as median (inter-quartiles range, IQR) and Student's *t*-test and Mann–Whitney *U* test, respectively, were applied to test differences between the groups.

For the continuous variables peak CKMB levels and prehospital time (in minutes) we conducted multiple imputation by chained equations (imputation method: “predictive mean matching” with linear regression as the regression model for the variables “peak CKMB levels” and “prehospital time”, the number of iterations: 5, number of created imputed data sets: 5). The variables were initially square-rooted due to a strong right-skewed distribution. The imputation process was performed with MICE-package (R statistic software). The subsequent regression models were calculated for each of the 5 imputed data sets and results were pooled in the end.

### 2.4 Logistic regression models

Logistic regression models were calculated in order to examine the association between diabetes and specific symptoms. First, unadjusted models were calculated. Next, we calculated models adjusted for sex and age. Finally, according to literature review, the multivariable adjusted logistic models were adjusted for sex, age, type of infarction (STEMI, NSTEMI, bundle branch block), renal function according to eGFR (groups: eGFR > 60 ml/min/1.73 m^2^, 30–60 ml/min/1.73 m^2^, < 30 ml/min/1.73 m^2^ and a group with missing information), left ventricular ejection fraction (groups: EF > 30%, EF ≤ 30%, no information on EF), prehospital time in minutes and peak CKMB levels. For the regression models, the values of continuous variables prehospital time and peak CKMB levels were used with square rooted values due to strong right-skewed distributions.

Moreover, we performed subgroup analysis to examine differences between age groups. We stratified the sample into 4 age groups: patients <55 years of age, patients between 55 and 64, patients between 65 and 74, and patient between 75 and 84 years. For each group, we calculated the multivariable adjusted logistic regression models as described above for the total sample.

All statistical analyses were performed using R version 4.2.1.

## 3 Results

Overall, 5,900 cases of first-time AMI were included in the present analysis. Table 1 displays baseline characteristics, symptoms at the event, clinical parameters and information about treatment stratified for diabetes (yes/no). In total, 1,859 (31.5%) patients had a known diagnosis of diabetes mellitus at the time of the acute event. Within the diabetes group, women were slightly overrepresented: 33.6% of all patients were female in the diabetes group, while in the non-diabetes group only 29.5% were women. With a mean age of 69.0 (SD: 10.7) years, the diabetes patients were significantly older than the non-diabetes patients with 65.1 (SD: 12.2) years. The patients with diabetes also had significantly more frequently other comorbidities (hypertension, hyperlipidemia, impaired renal function, severely impaired ventricular ejection fraction). On the other hand, patients with diabetes were significantly less likely to receive PCI treatment compared to non-diabetic AMI patients.

**Table 1:** Baseline characteristics for the total sample and stratified for diabetes (yes/no) given by total number and % or mean and SD or median and IQR.

	Diabetes (*n* = 1,859)	No diabetes (*n* = 4,041)	*P* value	*N* ^a^
Female	624 (33.6)	1,191 (29.5)	0.002	5,900
Age (mean, SD)	69.0 (10.7)	65.1 (12.2)	<0.001	5,900
28-day mortality (%)	143 (7.7)	229 (5.7)	0.004	5,900
Symptoms at the acute event				
Typical chest pain symptoms	1,327 (71.4)	3,222 (79.7)	<0.001	5,900
Pain—left arm/shoulder	674 (36.5)	1,764 (43.9)	<0.001	5,864
Pain—right arm/shoulder	345 (18.7)	1,005 (25)	<0.001	5,856
Pain—between shoulder blades	674 (38.9)	1,764 (47.2)	<0.001	5,470
Pain—upper abdomen	199 (10.8)	412 (10.3)	0.585	5,851
Pain—throat/jaw	331 (17.9)	881 (22.0)	<0.001	5,856
Sweating	702 (38.1)	1,795 (44.7)	<0.001	5,858
Vomiting/Nausea	494 (26.7)	1,265 (31.4)	<0.001	5,872
Shortness of breath	1,020 (55.1)	1,932 (48.0)	<0.001	5,877
Dizziness/Vertigo	332 (18.0)	855 (21.3)	0.004	5,857
Syncope/Unconsciousness	109 (5.9)	221 (5.5)	0.568	5,861
Fear of death/Feeling of annihilation	197 (10.7)	545 (13.6)	0.002	5,856
Comorbidities				
Number of years from diabetes diagnosis to AMI (median, IQR)	7.0 (1.0–15.0)	–	–	5,533
Hypertension	1,669 (89.8)	2,859 (70.7)	<0.001	5,900
Hyperlipidemia	1,145 (61.6)	1,962 (48.6)	<0.001	5,900
*Smoking status*			<0.001	5,900
Current smoker	419 (22.5)	1,377 (34.1)		
Never smoker	603 (32.4)	1,102 (27.3)		
Ex-smoker	629 (33.8)	1,239 (30.7)		
No information	208 (11.2)	323 (8)		
Clinical characteristics				
Prehospital time in minutes	169.5 (85.0–561.8)	155.0 (82.0–513.5)	0.133	4,565
Cardiac arrest (prehospital or during admission)	217 (11.7)	494 (12.2)	0.574	5,900
Peak CKMB levels (U/L)	58.0 (32.0–136.0)	78.0 (36.0–189.0)	<0.001	5,104
Troponin I (ng/ml)	0.7 (0.2–5.1)	0.7 (0.1–4.1)	0.120	2,846
*Type of infarction*			<0.001	5,900
STEMI	582 (31.3)	1,623 (40.2)		
NSTEMI	1,066 (57.3)	2,121 (52.5)		
Bundle branch block	211 (11.4)	297 (7.3)		
Heart rhythm at admission^b^			0.005	2,947
Sinus rhythm	814 (85.6)	1,790 (89.7)		
Atrial fibrillation	111 (11.7)	166 (8.3)		
Other/Unknown	26 (2.7)	40 (2.0)		
*Renal Function according to GFR*			<0.001	5,900
GFR ≥ 60 ml/min	1,008 (54.2)	2,886 (71.4)		
GFR 30–59 ml/min	633 (34.1)	965 (23.9)		
GFR < 30 ml/min	195 (10.5)	153 (3.8)		
No information	23 (1.2)	37 (0.9)		
Left ventricular ejection fraction			<0.001	5,900
≤30%	163 (8.8)	266 (6.6)		
>30%	1,472 (79.2)	3,432 (84.9)		
No information	224 (12.0)	343 (8.5)		
Treatment				
PCI	1,260 (67.8)	3,014 (74.6)	<0.001	5,900
Bypass therapy	269 (14.5)	507 (12.5)	0.047	5,900
Lysis therapy	3 (0.2)	28 (0.7)	0.015	5,900

^a^ Number of cases with valid information.

^b^ Information available for AMI cases from 2009 onwards.

### 3.1 Diabetes and symptoms at the acute event

The most frequently occurring symptom at the event was typical chest pain, which was reported by 71.4% of patients with diabetes, which is significantly less often than in the non-diabetes group (79.7%), see Table 1. The same refers to pain in other parts of the body, which occurred significantly more frequently in non-diabetes patients (except upper abdomen). Likewise, the unspecific symptoms sweating, vomiting/nausea, dizziness/vertigo and fear of death/feeling of annihilation were reported significantly less frequently by individuals with diabetes. Surprisingly, shortness of breath was the only symptom that was significantly more present in patients with diabetes (Table 1).

Table 2 shows the frequency of specific symptoms stratified for sex and diabetes. The figure indicates, that the associations between diabetes and various symptoms observed see Table 1 are valid for males and females alike.

**Table 2:** Symptoms of male and female patients at the acute event stratified for diabetes (yes/no).

	Female patients			Male patients		
	Diabetes (*n* = 624)	No diabetes (*n* = 1,191)	*P* value	Diabetes (*n* = 1,235)	No diabetes (*n* = 2,850)	*P* value
Symptoms at the acute event						
Typical chest pain symptoms	426 (68.3)	949 (79.7)	<0.001	901 (73.0)	2,273 (79.8)	<0.001
Pain—left arm/shoulder	235 (37.8)	553 (46.9)	<0.001	439 (35.8)	1,211 (42.7)	<0.001
Pain—right arm/shoulder	130 (21.0)	290 (24.6)	0.098	215 (17.6)	715 (25.2)	<0.001
Pain—between shoulder blades	235 (40.7)	553 (51.2)	<0.001	439 (38.0)	1,211 (45.6)	<0.001
Pain—throat/jaw	133 (21.3)	311 (26.1)	0.022	198 (16.0)	570 (20.0)	0.003
Pain—upper abdomen	65 (10.5)	144 (12.3)	0.301	134 (10.9)	268 (9.5)	0.163
Sweating	232 (37.4)	479 (40.7)	0.190	470 (38.5)	1,316 (46.4)	<0.001
Vomiting/Nausea	208 (33.3)	493 (41.6)	<0.001	286 (23.4)	772 (27.2)	0.012
Shortness of breath	365 (58.6)	620 (52.3)	0.012	655 (53.4)	1,312 (46.2)	<0.001
Dizziness/Vertigo	112 (18.0)	282 (23.9)	0.005	220 (18.0)	573 (20.2)	0.107
Syncope/Unconsciousness	33 (5.3)	87 (7.4)	0.118	76 (6.2)	134 (4.7)	0.059
Fear of death/Feeling of annihilation	71 (11.4)	181 (15.4)	0.027	126 (10.3)	364 (12.9)	0.024

**Figure 1:**
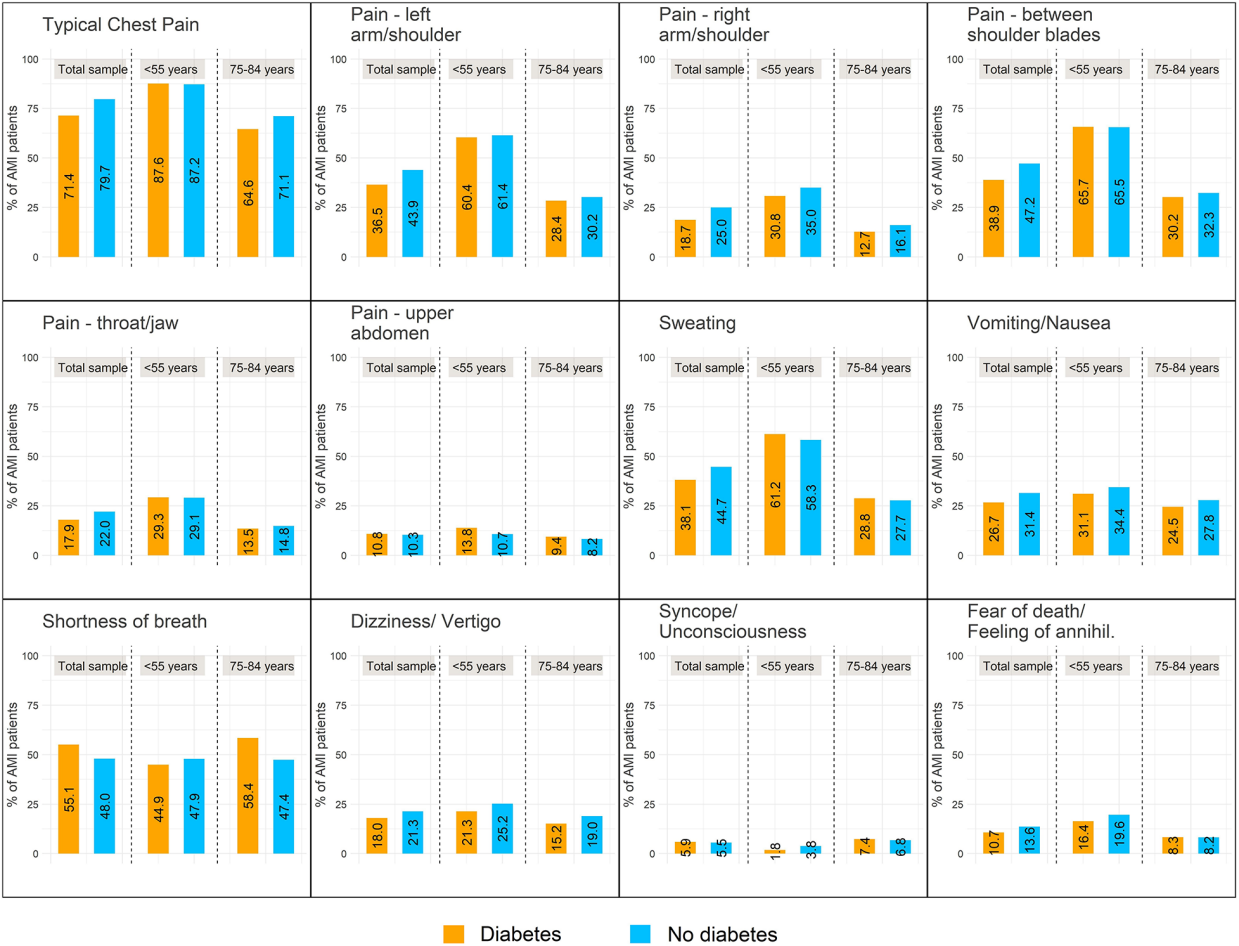
Relative frequencies of reported symptoms at AMI stratified for diabetes. On the left hand side of each plot the percentages for the total sample are plotted; in the middle the respective frequencies for young patients (<55 years) and on the right hand side the frequencies for older patients (age group 75–84 years) are displayed.

Figure 1 shows the percentages of different symptoms in the diabetes group and the non-diabetes group. The figure suggests, that the older age group does not differ very much from the total sample. But the figure also shows, that patients with diabetes in the age group 55 years and younger were more likely to have some specific symptoms compared to their non-diabetic controls indicating a different associations for this age group.

Table 3 displays the results of different logistic regression models analyzing the associations between diabetes (exposure) and specific symptoms (outcome). The unadjusted models and the models adjusted for sex and age actually provide comparable results in line with the relative frequencies shown in Table 1: while diabetes showed significant negative associations with several pain symptoms (including typical chest pain) and other symptoms, shortness of breath showed a strong positive association with diabetes. While the latter association between shortness of breath and diabetes was confirmed by the multivariable adjusted model, the association of diabetes with most other symptoms appeared strongly attenuated in the multivariable adjusted model (only typical chest pain symptoms and pain in the right arm/shoulder remained significant).

**Table 3:** Association between diabetes and specific symptoms at the acute event analyzed by different logistic regression models: unadjusted model (left), model adjusted for sex and age (middle) and multivariable adjusted model.

	Unadjusted model		Adjusted for sex an age		Multivariable adjusted model^a^	
Symptoms at the acute event	OR [95%CI]	*p* value	OR [95%CI]	*p* value	OR [95%CI]	*p* value
Typical chest pain symptoms	0.63 [0.56, 0.72]	<0.001	0.71 [0.62, 0.80]	<0.001	0.84 [0.73, 0.96]	0.012
Pain—left arm/shoulder	0.73 [0.66, 0.82]	<0.001	0.84 [0.75, 0.95]	0.005	0.94 [0.83, 1.06]	0.312
Pain—right arm/shoulder	0.69 [0.60, 0.79]	<0.001	0.78 [0.68,0.90]	<0.001	0.85 [0.74, 0.99]	0.031
Pain—between shoulder blades	0.71 [0.63, 0.80]	<0.001	0.83 [0.74, 0.94]	0.003	0.93 [0.82, 1.06]	0.272
Pain—throat/jaw	0.77 [0.67, 0.89]	<0.001	0.86 [0.74, 0.99]	0.034	0.91 [0.79, 1.06]	0.231
Pain—upper abdomen	1.06 [0.88, 1.26]	0.554	1.08 [0.90, 1.30]	0.391	1.12 [0.93, 1.35]	0.216
Sweating	0.76 [0.68, 0.85]	<0.001	0.88 [0.79, 0.99]	0.035	0.98 [0.87, 1.10]	0.724
Vomiting/Nausea	0.80 [0.70, 0.90]	<0.001	0.82 [0.72, 0.93]	0.002	0.89 [0.78, 1.01]	0.082
Shortness of breath	1.33 [1.19, 1.49]	<0.001	1.31 [1.17, 1.47]	<0.001	1.24 [1.10, 1.39]	<0.001
Dizziness/Vertigo	0.81 [0.70, 0.93]	0.003	0.85 [0.74, 0.98]	0.029	0.89 [0.77, 1.03]	0.119
Syncope/Unconsciousness	1.08 [0.85, 1.37]	0.528	0.99 [0.78, 1.26]	0.932	0.94 [0.74, 1.21]	0.643
Fear of death/Feeling of annihilation	0.76 [0.64, 0.90]	0.002	0.85 [0.71, 1.01]	0.064	0.91 [0.76, 1.09]	0.296

^a^ Adjusted for sex, age, type of infarction (STEMI, NSTEMI, bundle branch block), renal function according to GFR, severely impaired left ventricular ejection fraction, prehospital time, peak CKMB levels.

The subgroup analysis for different age groups (see Table 4) revealed that the above reported associations are largely not seen in patients below age 55 [no significant associations were found for this age group and the obtained results (point estimators/odds ratio) pointed in the opposite direction for some symptoms like shortness of breath]. In terms of a sensitivity analysis, we did the same calculations with only three age groups (<65 years, 65–79 years and ≥80 years), see Supplementary Table S1. It shows, that indeed only the very young patients <55 years differ substantially from the other age groups, since the results for the group <65 years are quite comparable to the age group 65–79 years.

**Table 4:** Association between diabetes and specific symptoms at the acute event analyzed by multivariable adjusted logistic regression models^a^ and stratified for different age groups.

	Age <55 years (*n* = 1,140)		Age 55–64 years (*n* = 1,275)		Age 65–74 years (*n* = 1,690)		Age 75–84 years (*n* = 1,795)	
Symptoms at the acute event	OR [95%CI]	*p* value	OR [95%CI]	*p* value	OR [95%CI]	*p* value	OR [95%CI]	*p* value
Typical chest pain symptoms	1.19 [0.75, 1.90]	0.459	0.80 [0.57, 1.10]	0.172	0.76 [0.59, 0.97]	0.031	0.87 [0.70, 1.08]	0.201
Pain—left arm/shoulder	1.04 [0.76, 1.42]	0.796	0.69 [0.53, 0.90]	0.007	1.01 [0.81, 1.26]	0.906	1.08 [0.87, 1.35]	0.491
Pain—right arm/shoulder	0.86 [0.63, 1.19]	0.360	0.86 [0.63, 1.17]	0.337	0.86 [0.67, 1.11]	0.258	0.87 [0.65, 1.16]	0.346
Pain—between shoulder blades	1.10 [0.79, 1.53]	0.579	0.69 [0.52, 0.91]	0.009	0.97 [0.78, 1.22]	0.811	1.06 [0.85, 1.33]	0.592
Pain—throat/jaw	1.01 [0.73, 1.41]	0.930	0.97 [0.71, 1.33]	0.872	0.79 [0.60, 1.03]	0.085	1.00 [0.75, 1.33]	0.990
Pain—upper abdomen	1.30 [0.83, 2.03]	0.246	0.81 [0.52, 1.27]	0.367	1.12 [0.81, 1.54]	0.495	1.22 [0.87, 1.72]	0.257
Sweating	1.21 [0.89, 1.65]	0.220	0.87 [0.67, 1.14]	0.314	0.79 [0.64, 0.99]	0.037	1.17 [0.94, 1.46]	0.153
Vomiting/Nausea	0.85 [0.61, 1.17]	0.313	0.92 [0.69, 1.23]	0.591	0.83 [0.66, 1.05]	0.122	0.92 [0.73, 1.15]	0.458
Shortness of breath	0.85 [0.63, 1.15]	0.291	1.29 [0.99, 1.68]	0.058	1.23 [0.99, 1.54]	0.061	1.46 [1.20, 1.78]	<0.001
Dizziness/Vertigo	0.84 [0.58, 1.20]	0.332	0.92 [0.67, 1.26]	0.610	0.99 [0.76, 1.30]	0.966	0.81 [0.62, 1.05]	0.107
Syncope/Unconsciousness	0.48 [0.17, 1.39]	0.178	0.81 [0.44, 1.48]	0.488	1.00 [0.64, 1.56]	0.997	1.07 [0.73, 1.57]	0.722
Fear of death/Feeling of annihilation	0.82 [0.55, 1.22]	0.331	0.80 [0.54, 1.18]	0.267	0.90 [0.64, 1.26]	0.530	1.09 [0.77, 1.56]	0.625

^a^ Adjusted for sex, age, type of infarction (STEMI, NSTEMI, bundle branch block), renal function according to GFR, severely impaired left ventricular ejection fraction, prehospital time, peak CKMB levels.

## 4 Discussion

The present study shows that AMI patients with diabetes experience less often typical AMI symptoms, including the most characteristic symptom, i.e., chest pain. Only shortness of breath was reported more frequently by diabetic patients. Nevertheless, adjusting for several potential confounders the overall associations appeared to be attenuated, but remained significant for chest pain (more often in patients without diabetes) and shortness of breath (more often in patients with diabetes). Further analyses revealed, that the observed associations did not apply to younger AMI patients (age 54 and younger) with no significant associations between diabetes and specific symptoms at all.

Also prior studies have reported that diabetes goes along with less AMI symptoms including typical chest pain as well (5–10). A current review/meta-analysis specifically analyzing chest pain symptoms and diabetes came to the same conclusion (17). However, there were also studies finding no significant differences in reported AMI symptoms between patients with and without diabetes or even some atypical symptoms more frequently reported by diabetic patients (11–15). In particular, a study from Sweden (Northern Sweden MONICA Study) analyzed 4,028 patients with first myocardial infarction aged 25–74 years (14). They found no difference regarding typical symptoms between patients with and without diabetes, and diabetes was not identified as a predictor of atypical symptoms. So, this large study based on a myocardial infarction registry data came to differing results compared to the results in the present analysis. It can be speculated though, whether the different time frames of patient recruitment (between 2000 and 2006 for the Swedish study and 2010–2017 for the present study) had any influence on the results (e.g., the wide-spread use of high-sensitive troponin diagnostics led to the diagnosis of more smaller infarctions with higher frequencies of atypical symptoms).

Most studies reporting on this specific topic have not applied multivariable-adjusted regression models. So, their findings cannot be interpreted in the way that diabetes is independently associated with less or atypical AMI symptoms. And indeed, the results of the present study suggested that even though some associations remain significant, the estimated effects were considerably attenuated by the multivariable adjustment. In a sensitivity analysis we calculated a variety of different logistic regression models with varying adjusting variables. It was found, that in particular the inclusion of the variables eGFR group (representing renal function) and left ventricular ejection fraction into the regression models affected the results [attenuation of the point estimators (Odds ratio) for diabetes]. These observations are confirmed by a study from Korea which identified renal dysfunction as a major risk factor for painless AMI (18). The authors concluded, that particularly the joint occurrence of reduced GFR and diabetes mellitus went along with an increased appearance of painless AMI.

In the present study, there was a positive association between shortness of breath at acute MI and diabetes, which was the only significant positive association we found. This association remained significant in the multivariable adjusted logistic regression model for the total sample, but could not be detected in the youngest age group however. Prior studies have also indicated that patients with diabetes experience more frequently some form of shortness of breath or hyperventilation (6, 7, 11).

One major pathophysiological mechanisms underlying the observed phenomenon might be the impact of diabetic polyneuropathy as a complication of long-standing diabetes. Polyneuropathy is a micro-damage of the peripheral nerve system and caused by (chronic) hyperglycemia and altered insulin signaling (19). With up to 50% of all diabetic patients it is a very common complication and the prevalence rises with disease duration (19). Especially in diabetic patients with confirmed coronary artery disease the prevalence seems to be particularly high with a majority of those patients suffering from a polyneuropathy (19). Diabetic polyneuropathy quite frequently goes along with a decreased pain perception (19), which may be responsible for the reduced (pain) symptoms in diabetic AMI patients. However, in most cases it takes years or decades for a relevant diabetic polyneuropathy to develop. So, the diabetes diseases must preexist the acute event for quite a long time period. In fact, this would fit well into the picture that emerged when analyzing the different age groups: we found no reduced frequency of (pain) symptoms in young diabetes patients. These patients simple may be too young to have developed major complications like polyneuropathy. It may become a major issue in older age for which we found reduced frequencies of major (pain) symptoms.

We cannot explain why diabetes was strongly positively associated with shortness of breath; but again not in young patients however. Shortness of breath is a pretty unspecific symptom that cannot only occur in AMI patients as part of the typical angina pectoris symptom, but is also frequent in many other diseases like pulmonary embolism or pneumonia. It often also accompanies exceptional psychological situation like panic attacks and severe states of anxiety. With all this said, shortness of breath might also, at least in some sense, represent the patient’s current cardiovascular condition, e.g., in terms of acute heart failure with dyspnea as the leading symptom (20). Actually it has been reported that the presence of shortness of breath is associated with higher mortality after AMI (21). So it could be speculated if this particular symptom might be somehow an expression of more severe events, worse cardiovascular status and an overall worse prognosis; a condition for which diabetes is a well-known (causal) risk factor.

The results of this analysis underline the importance of thorough diagnostics in AMI-suspected patients with diabetes even without typical chest pain symptoms, which means an ECG within 10 min to rule out STEMI and serial high-sensitive Troponin testing to detect NSTEMI (22). Especially in older patients with diabetes, AMIs might be hidden under rather unspecific symptoms, like shortness of breath. The absence of typical AMI symptoms, in particular chest pain, goes along with an increased risk of delayed diagnosis and treatment (23–25), which can lead to an elevated mortality and overall worse outcomes (10, 21, 23–26). Accordingly, AMI patients with diabetes indeed have longer pre-hospital delays (13, 15, 27) and a higher mortality after the event (3, 4). Awareness of this particularity in clinical presentation is key to a fast diagnosis and subsequent treatment, which is especially important in patients with the additional risk factor diabetes mellitus.

### 4.1 Strengths and limitations

The present study is characterized by some major strengths. The data analyzed was collected by a population-based registry, avoiding any selection bias as best as possible. The relatively high number of patients included into this analysis provides solid statistical power. Symptoms at the acute event were assessed by a standardized questionnaire in a face-to-face interview only days after the infarction. For each patients, a variety of different information and data was collected that allowed to perform multivariable adjusted logistic regression models.

There are also some limitations. First, we have no patients older than 85 years included into this study. Likewise, no information on ethnicity was available, so the results might not be generalized to all ethnicities and age groups. Patients that were unable or unwilling to complete the interview and report their symptoms could not be considered for this study and their absence in the analyses might have affected the overall results. The interview with the AMI patients was conducted several days after admission, so fading memory might have affected the reporting of symptoms in some cases. Since there is an unneglectable number of AMI patients with undiagnosed diabetes (28), we might have misclassified some patients as non-diabetes patients. Finally, since some potentially important information (for instance on pulmonary pathologies) were missing, we might have not have considered all relevant confounders in the multivariable adjusted models.

## 5 Conclusion

AMI patients with diabetes have less symptoms at the acute event compared to patients without diabetes. One major exception is shortness of breath, which is significantly more often present in diabetic patients. However, the results obtained for the total sample could not be reproduced in patients younger than 55 years.

This studies demonstrates that patients with diabetes often report less symptoms at the acute event despite an overall worse prognosis. Especially the absence of typical chest pain and the presence of shortness of breath might be typical for diabetes in AMI patients aged 55+. Consequently, fast diagnostics and quick treatment appears to be essential particularly in these patients.

## Data Availability

The raw data supporting the conclusions of this article will be made available by the authors, without undue reservation.
