# Supplementary material for: Do patients with diabetes with new onset acute myocardial infarction present with different symptoms than non-diabetic patients?
Source: Front Cardiovasc Med. 2024 Jan 15;11:1324451. doi: 10.3389/fcvm.2024.1324451 (PMC10822885; doi:10.3389/fcvm.2024.1324451)
Supplement: Supplementary file 1 [file Table1.pdf]

Supplementary material

Do patients with diabetes with new onset acute myocardial infarction present with different symptoms than non-diabetic patients?

Timo Schmitz, Bastian Wein, Philip Raake, Margit, Annette Peters, Jakob Linseisen, Christa Meisinger

Table S1: Association between diabetes and specific symptoms at the acute event analyzed by multivariable adjusted logistic regression models* and stratified for the age groups <65 years, 65-79 years and ≥ 80 years.

	Age <65 years *(n=2415)*		***Age 65-79 years*** *(n=2657)*		***Age ≥ 80 years*** *(n=828)*	
**Symptoms at the acute event**	***OR [95%CI]***	***p value***	***OR [95%CI]***	***p value***	***OR [95%CI]***	***p value***
Typical chest pain symptoms	0.91 [0.70,1.19]	0.504	0.78 [0.64,0.94]	0.010	0.92 [0.68,1.26]	0.614
Pain - left arm / shoulder	0.83 [0.68,1.01]	0.061	1.01 [0.85,1.21]	0.886	1.15 [0.83,1.61]	0.395
Pain - right arm / shoulder	0.85 [0.69,1.06]	0.160	0.83 [0.67,1.02]	0.079	1.01 [0.66,1.54]	0.977
Pain - between shoulder blades	0.84 [0.68,1.04]	0.104	0.98 [0.82,1.18]	0.862	1.11 [0.79,1.55]	0.546
Pain - throat / jaw	0.98 [0.78,1.22]	0.830	0.86 [0.69,1.07]	0.183	0.93 [0.59,1.45]	0.737
Pain - upper abdomen	1.04 [0.76,1.42]	0.796	1.11 [0.85,1.44]	0.440	1.41 [0.85,2.34]	0.182
Sweating	1.00 [0.82,1.22]	0.977	0.92 [0.78,1.10]	0.360	1.07 [0.77,1.51]	0.678
Vomiting/Nausea	0.90 [0.73,1.12]	0.338	0.84 [0.69,1.01]	0.057	1.00 [0.71,1.41]	0.999
Shortness of breath	1.09 [0.89,1.32]	0.405	1.35 [1.14,1.59]	<0.001	1.27 [0.95,1.71]	0.109
Dizziness/ Vertigo	0.90 [0.71,1.14]	0.394	0.92 [0.75,1.14]	0.452	0.76 [0.51,1.13]	0.177
Syncope/ Unconsciousness	0.71 [0.43,1.18]	0.190	1.08 [0.76,1.51]	0.676	0.99 [0.58,1.69]	0.965
Fear of death / Feeling of annihilation	0.83 [0.63,1.09]	0.176	0.92 [0.70,1.20]	0.526	1.26 [0.73,2.18]	0.413

* adjusted for sex, age, type of infarction (STEMI, NSTEMI, bundle branch block), renal function according to GFR, severely impaired left ventricular ejection fraction, prehospital time, peak CKMB levels
